# Utility of the Framingham risk score to predict the presence of coronary atherosclerosis in patients with rheumatoid arthritis

**DOI:** 10.1186/ar2098

**Published:** 2006-12-14

**Authors:** Cecilia P Chung, Annette Oeser, Ingrid Avalos, Tebeb Gebretsadik, Ayumi Shintani, Paolo Raggi, Tuulikki Sokka, Theodore Pincus, C Michael Stein

**Affiliations:** 1 Department of Medicine, Vanderbilt University School of Medicine, 1161 21st Ave., Nashville, TN 37232, USA; 2 Department of Biostatistics, Vanderbilt University School of Medicine, S-2323 Medical Center North, Nashville, TN 37232, USA; 3 Department of Medicine, Division of Cardiology, Emory University School of Medicine, 1365 Clifton Road NE, AT-504, Atlanta, GA 30322; 4 Jyväskylä Central Hospital, Keskussairaalantie 19, 40620 Jyväskylä, Finland; 5 Department of Pharmacology, Vanderbilt University School of Medicine, 542 RRB, Nashville, TN 37232, USA

## Abstract

The prevalence of ischemic heart disease and atherosclerosis is increased in patients with rheumatoid arthritis (RA). In the general population, but not in patients with systemic lupus erythematosus, the Framingham risk score identifies patients at increased cardiovascular risk and helps determine the need for preventive interventions. We examined the hypothesis that the Framingham score is increased and associated with coronary-artery atherosclerosis in patients with RA. The Framingham score and the 10-year cardiovascular risk were compared among 155 patients with RA (89 with early disease, 66 with long-standing disease) and 85 control subjects. The presence of coronary-artery calcification was determined by electron-beam computed tomography. The Framingham score was compared in patients with RA and control subjects, and the association between the risk score and coronary-artery calcification was examined in patients. Patients with long-standing RA had a higher Framingham score (14 [11 to 18]) (median [interquartile range]) compared to patients with early RA (11 [8 to 14]) or control subjects (12 [7 to 14], *P *< 0.001). This remained significant after adjustment for age and gender (*P *= 0.015). Seventy-six patients with RA had coronary calcification; their Framingham risk score was higher (14 [12 to 17]) than that of 79 patients without calcification (10 [5 to 14]) (*P *< 0.001). Furthermore, a higher Framingham score was associated with a higher calcium score (odds ratio [OR] = 1.20, 95% confidence interval [CI] 1.12 to 1.29, *P *< 0.001), and the association remained significant after adjustment for age and gender (OR = 1.15, 95% CI 1.02 to 1.29, *P *= 0.03). In conclusion, a higher Framingham risk score is independently associated with the presence of coronary calcification in patients with RA.

## Introduction

Patients with rheumatoid arthritis (RA) have increased mortality, largely attributable to cardiovascular disease [1], and there is increasing evidence from controlled clinical studies that patients with RA have more extensive extra-coronary atherosclerosis [2-5] and coronary calcification [6] than age- and gender-matched control subjects. The contribution of traditional and novel cardiovascular risk factors has been examined, but the mechanism for this increased cardiovascular risk in RA remains unclear.

Hypertension, smoking, and increased concentrations of C-reactive protein are more frequent in patients with RA than in control subjects [7,8]. Furthermore, patients with RA and coronary-artery atherosclerosis are older and have a higher cumulative exposure to cigarettes and a higher erythrocyte sedimentation rate than patients without atherosclerosis, suggesting that traditional risk factors and inflammation may both play a role in the process [6].

The Framingham risk score is an extensively studied index to predict cardiovascular risk in the general population [9]. It includes age, gender, smoking, blood pressure, and cholesterol concentrations and estimates the risk of coronary events by stratifying individuals into three risk categories: low (<10% risk of an event in 10 years), intermediate (10% to 20%), and high (>20%) [10]. Although the Framingham risk score is widely used in the general population to determine prognosis and the need for intervention [9], the value of this risk score is less clear in younger patients, women, and patients with inflammatory diseases [11,12]. For example, in patients with systemic lupus erythematosus (SLE), another rheumatic disease associated with increased coronary calcification, there were no differences in the Framingham risk score in patients and controls, and the majority of the SLE patients with coronary calcification had a low 10-year risk according to the Framingham calculations [12].

In contrast to the general population, there is no information about the relationship between the Framingham cardiovascular risk score and coronary calcification in patients with RA. Therefore, we tested the hypothesis that the Framingham cardiovascular risk score would be associated with coronary calcification in patients with RA.

## Materials and methods

Eighty-nine patients with early RA who had a median (interquartile range) disease duration of 2 (1 to 3) years and mean age of 51 ± 11 (mean ± standard deviation) years, 66 patients with long-standing RA (median disease duration 19 [14 to 24] years), aged 57 ± 10 years, who met the classification criteria for RA [13], and 85 control subjects aged 52 ± 10 years were studied. Control subjects did not meet the classification criteria for RA or any other autoimmune disease and were frequency-matched for age, gender, and race with patients with RA (early and long-standing disease combined). These patients and control subjects are part of an ongoing cohort study to identify cardiovascular risk factors in RA [6]. The study was approved by the Institutional Review Board of Vanderbilt University Hospital, and all subjects gave written informed consent.

### Clinical assessment

Patients and control subjects were assessed according to a standardized clinical interview, physical examination, laboratory tests, and (in patients) chart review. Family history of coronary disease was defined as a first-degree relative who had had a myocardial infarction or stroke before the age of 55 in males or 65 in females. Height and weight were measured, and body mass index was calculated by dividing the weight in kilograms by the square of the height in meters. Blood pressure was recorded as the mean of two measurements obtained 5 minutes apart after subjects had rested in a supine position for 10 minutes.

### Laboratory tests

After an overnight fast, blood was collected for measurement of glucose, total cholesterol, high-density lipoprotein (HDL), triglycerides, Lp(a) lipoprotein, and homocysteine concentrations and the calculation of low-density lipoprotein (LDL).

### RA measurements of disease activity and other outcomes

In patients with RA, disease activity was measured with the use of the disease activity score using 28 joint counts (DAS28) [14]. All patients and control subjects completed a Modified Health Assessment Questionnaire that provided scores for physical function, pain (0 to 10 cm, visual analog scale), and fatigue (visual analog scale) [15].

### Framingham score

The composite simplified coronary prediction model built on the blood pressure and cholesterol categories proposed by the Joint National Committee on Blood Pressure and the National Cholesterol Education Program was used. This model includes age, total and HDL cholesterol, blood pressure, and smoking and was designed in the setting of a community-based cohort (Framingham) of more than 5,000 people followed for 12 years [9].

### Coronary-artery calcification

All subjects underwent imaging with an Imatron C-150 scanner (GE Imatron, now part of GE Healthcare, Little Chalfont, Buckinghamshire, UK) as described previously [6]. Briefly, imaging was performed with a 100-millisecond scanning time and a single-slice thickness of 3 mm. Forty slices were obtained during a single breath-holding period starting at the aortic arch and proceeding to the level of the diaphragm. Tomographic imaging was electrocardiographically triggered at 60% of the interval between R waves. All areas of calcification within the borders of a coronary artery with a minimal attenuation of 130 Hounsfield units (HU) were computed. A calcified coronary plaque was considered present if at least three contiguous pixels were detected (voxel size, 1.03 mm^3^). All the scans were read by a single expert investigator (PR), unaware of the subjects' clinical status.

### Calculation of calcium scores

The degree of coronary-artery calcification was calculated as described by Agatston *et al*. [16]. The area of each calcified plaque is multiplied by the peak radiological attenuation inside this area expressed as a coefficient (1 = for an attenuation of 130 to 199 HU; 2 = 200 to 299 HU; 3 = 300 to 400 HU; and 4 = more than 400 HU). The sum of the scores for all coronary arterial lesions provides an overall score for each individual.

### Statistical analysis

Clinical characteristics and the cardiovascular risk scores were compared in patients with early RA, patients with long-standing RA, and control subjects using Kruskal-Wallis or Wilcoxon rank sum tests for continuous variables and Fisher's exact test for categorical variables. The correlation between Framingham score and continuous clinical characteristics was performed using the Spearman correlation coefficient (ρ). A multiple linear regression model with adjustment for age and gender was used to assess the difference in the Framingham score among control subjects and patients with early and long-standing RA.

Coronary calcium scores were classified as absent (0), minimal to mild (1 to 100), moderate (101 to 400), and severe (>400), and the association between these categories and the risk score was examined by proportional logistic regression in patients with RA and control subjects. In the multivariable analyses, non-linear effect of age was evaluated using a restricted cubic spline [17]. All analyses used a 5% two-sided significance level and were performed with STATA 9.1 and R 2.1.0 (STATACorp, College Station, TX, USA) [18].

## Results

Demographic characteristics and cardiovascular risk factors of patients with RA and control subjects are shown in Table 1. Patients with RA had a median DAS28 of 3.29 (2.26 to 4.29), median C-reactive protein of 4 (3 to 11) mg/l, and median erythrocyte sedimentation rate of 15 (7 to 35) mm/hour. One hundred eleven (72%) were current users of methotrexate. As reported previously [6], several traditional cardiovascular risk factors differed among the three groups, including individual components of the Framingham equation such as age (*P *= 0.002), percentage of current smokers (*P *= 0.004), and systolic blood pressure (*P *= 0.001). Serum concentrations of total and HDL cholesterol were not statistically significant (*P *= 0.22 and *P *= 0.16, respectively). Patients with long-standing disease had higher Framingham risk scores (14 [11 to 18] units) than patients with early disease (11 [8 to 14] units) or control subjects (12 [7 to 14] units) (*P *< 0.001). The association for long-standing RA versus control subjects remained significant after statistical adjustment for age and gender (β = 1.38 [95% confidence interval (CI) 0.26 to 2.49], *P *= 0.015). These results remained significant after further adjustment for homocysteine (β = 1.36 [95% CI 0.24 to 2.49], *P *= 0.018).

**Figure 1:**
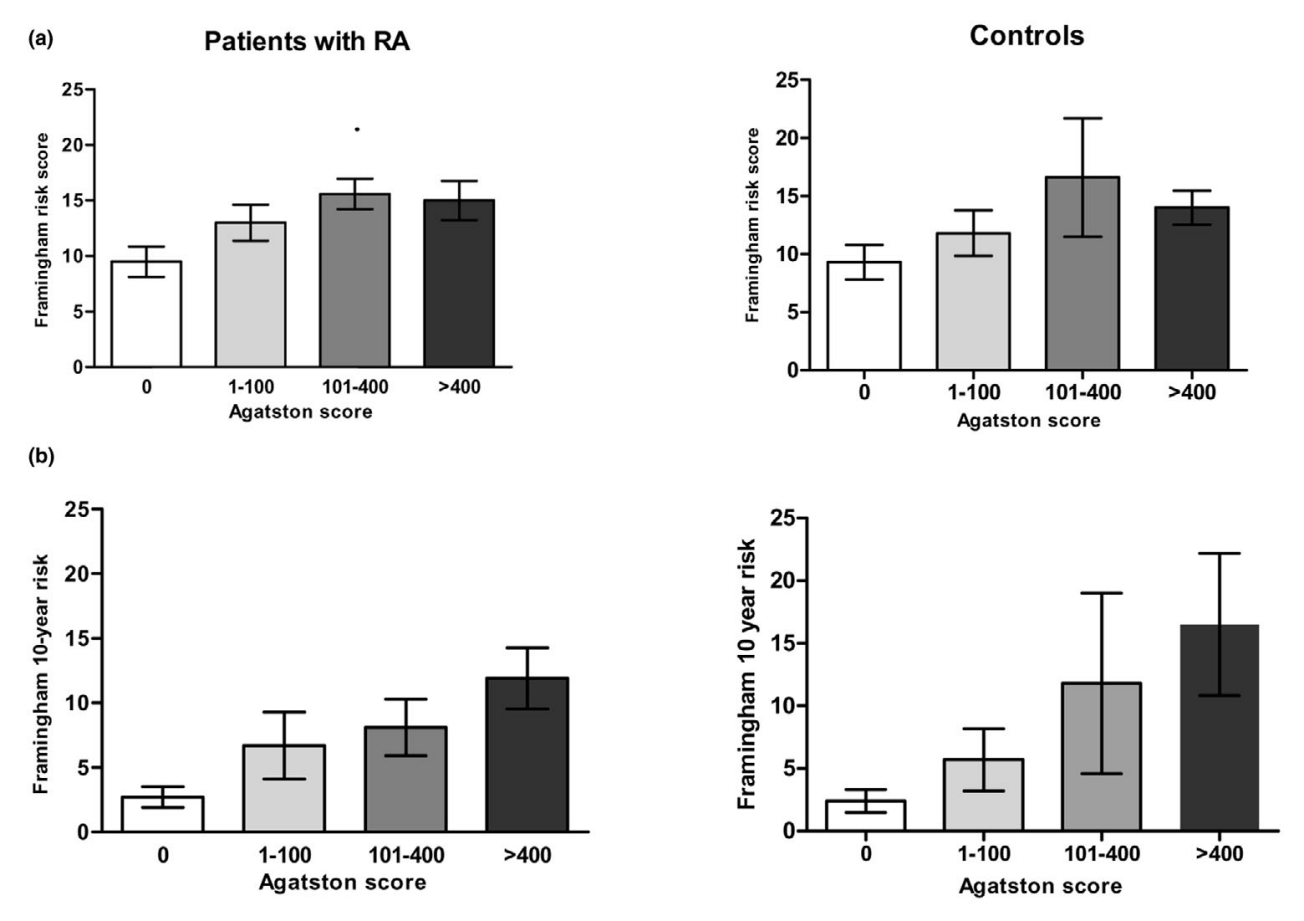
Framingham risk score, the calculated 10-year risk of a cardiovascular event, and coronary-artery calcification of varying severity in patients with rheumatoid arthritis (RA) and control subjects. Framingham score **(a) **and the calculated 10-year risk of a cardiovascular event **(b) **and coronary-artery calcification of varying severity in patients with RA and control subjects. Error bars represent mean and 95% confidence interval.

Framingham scores were significantly higher (14 [12 to 17] units) in 76 patients with RA who had coronary-artery calcification compared with 79 patients who did not have coronary calcification (10 [5 to 14] units) (*P *< 0.001). Figure 1 shows the Framingham risk score and the 10-year risk according to coronary calcification categories. The Framingham risk scores were correlated with Agatston scores (ρ = 0.44, *P *< 0.001), and an increase in one unit of the Framingham risk score was associated with a 20% increase in the odds of higher Agatston scores (odds ratio [OR] = 1.20, 95% CI 1.12 to 1.29, *P *< 0.001). This association remained significant after adjustment for age and gender (OR = 1.15, 95% CI 1.02 to 1.29, *P *= 0.03) and after further adjustment for homocysteine concentrations (OR = 1.15, 95% CI 1.01 to 1.30, *P *= 0.03). Similar results were found in control subjects, in whom the association between the Framingham score and coronary calcium score was significant (OR = 1.18, 95% CI 1.07 to 1.31, *P *< 0.001), and remained independent of age, gender, systolic blood pressure, and homocysteine (OR = 1.20, 95% CI 1.02 to 1.41, *P *= 0.03). However, the association of the Framingham risk score and coronary calcification is not modified by disease status (interaction analysis, *P *= 0.48), suggesting that the Framingham risk score has similar predictive ability among patients with RA and control subjects.

Patients with coronary calcification also had a higher predicted 10-year Framingham risk of a cardiovascular event (7% [4% to 12%]) than patients who did not have calcification (1% [<1% to 4%]) (*P *< 0.001). Table 2 shows the correlations between the Framingham risk score and clinical characteristics and cardiovascular risk factors among patients with RA. As expected, the score was significantly correlated with its individual components: age (ρ = 0.76, *P *< 0.001), systolic blood pressure (ρ = 0.60, *P *< 0.001), and total cholesterol (ρ = 0.27, *P *< 0.001). In addition, other cardiovascular risk factors such as concentrations of triglycerides (ρ = 0.26, *P *= 0.001), diastolic blood pressure (ρ = 0.22, *P *= 0.006), and LDL cholesterol (ρ = 0.22, *P *= 0.005) were also significantly correlated with the Framingham score. Some specific disease characteristics, including disease duration (ρ = 0.29, *P *< 0.001) and cumulative corticosteroids (ρ = 0.17, *P *= 0.03), were correlated significantly with the Framingham score, but cumulative hydroxychloroquine (*P *= 0.53) and functional capacity (*P *= 0.90) were not. An association with DAS28 was of borderline significance (*P *= 0.07).

**Figure 2:**
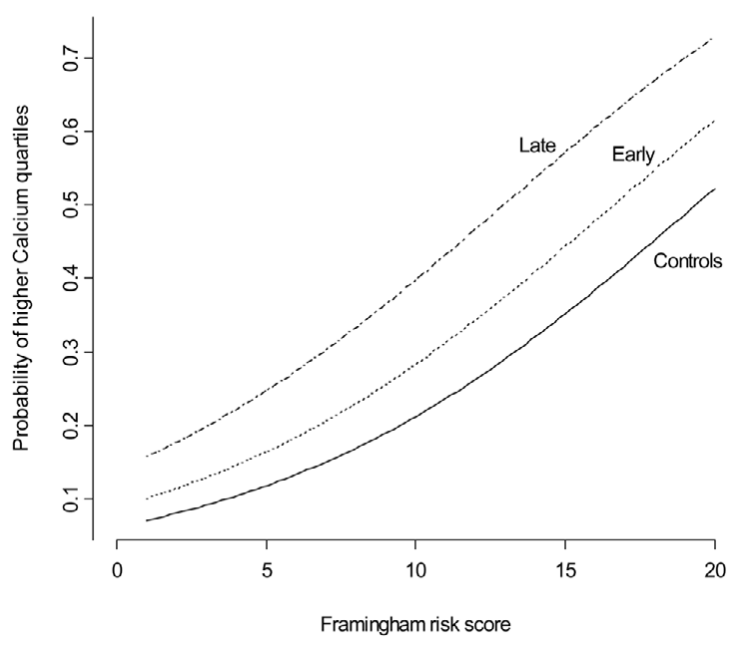
Relationship between the Framingham risk score and the probability of higher calcium scores among patients with early and long-standing rheumatoid arthritis (RA) and control subjects. The results of a multivariable model examining the association of the Framingham risk and coronary calcium scores after adjusting for disease status, age, gender, and homocysteine are depicted. Due to differences in the intercept, patients with long-standing RA (late) have a greater probability of higher coronary calcium scores than control subjects, independent of Framingham risk scores (odds ratio = 2.46, 95% confidence interval 1.20 to 5.05).

Figure 2 depicts the results of a multivariable model examining the association of the Framingham risk and coronary calcium scores after adjusting for disease status, age, gender, and homocysteine. Differences in the intercept indicate that patients with long-standing RA (late) have a greater probability of having higher coronary calcium scores than control subjects, independent of Framingham risk scores (OR = 2.46, 95% CI 1.20 to 5.05).

The control group was frequency-matched for age and gender with the entire group of RA patients and thus predictably did not exactly match with either the early or late RA groups. Minor differences between the groups were dealt with by the use of multivariable adjusted analyses. To further confirm our results, we performed sensitivity analyses between all patients with long-standing RA (mean age 56.7 ± 10.5 years, 25.8% male) and 66 matched control subjects (mean age 55.0 ± 7.6 years, 25.8% male). The association between RA and Framingham score was of borderline significance (β = 1.62, 95% CI -0.01 to 3.26, *P *= 0.05) and remained significant after adjusting for age, gender, and homocysteine (β = 1.40, 95% CI 0.17 to 2.64, *P *= 0.03). Also, the association between the Framingham risk and coronary calcium scores was significant (unadjusted OR = 1.16, 95% CI 1.07 to 1.25, *P *< 0.001) and remained so after adjustment for age, gender, and homocysteine (OR = 1.21, 95% CI 1.08 to 1.35, *P *= 0.001).

## Discussion

The two primary findings in this report are that patients with long-standing RA had higher Framingham risk scores compared with patients with early disease and control subjects and that patients with coronary calcification also have higher Framingham scores than those without coronary calcification.

The Framingham risk score includes age, gender, total and HDL cholesterol, blood pressure, diabetes, and smoking to derive an estimated risk of developing coronary heart disease within 10 years [9]. However, despite widespread use, the guidelines underestimate the presence of coronary calcification in certain populations, particularly women [11]. Because RA affects predominantly women, the utility of the Framingham risk score in this patient population is of interest, particularly considering that in patients with SLE, the Framingham risk score does not differ among patients and control subjects, and is inadequate for cardiovascular risk stratification [12,19].

Smoking and hypertension, two of the components of the Framingham risk score, differed among patients with early RA, patients with long-standing RA, and control subjects. There were more smokers among patients with early and late RA than among control subjects, and patients with late disease had higher systolic blood pressure. These differences explain in part why patients with long-standing disease had higher risk scores after adjustment for age and gender. This finding appears to be important as both risk factors can be modified, thus emphasizing the need for behavioral and therapeutic interventions and better control of common comorbidities in patients with RA.

The association between the Framingham risk score and additional cardiovascular risk factors and RA-specific characteristics such as cumulative exposure to corticosteroids and disease duration is of interest, as is the association between the Framingham risk score and the severity of coronary calcification in patients with RA. However, although the Framingham score and the 10-year risk estimates were higher in patients with coronary calcification, the majority of patients with coronary calcification were classified as being at 'low' 10-year risk and only 29 of 76 patients (38%) were at moderate or high risk according to the Framingham risk score. To improve prediction of future cardiovascular events in the general population, novel risk factors such as coronary calcium score have been studied.

A meta-analysis including asymptomatic individuals indicated that those with coronary-artery calcification above the median have an 8.7-fold increased risk of future coronary events [20]. In addition, there are data indicating that progression in calcium scores is associated with higher risk of myocardial infarction [21], and coronary-artery calcification adds information to the prediction of overall mortality [22]. Thus, efforts have been made to improve the prediction of risk by combining the Framingham risk score with coronary calcium score, and this combination appears of value to estimate risk in the general population [23,24]. In RA patients with coronary calcification, the use of such a modified Framingham score would increase the estimation of 10-year risk from a mean of 7% (4% to 12%) to 12% (5% to 22%) and 51 of 76 patients with coronary calcification would be classified as being at moderate to high risk and therefore would be candidates for aggressive therapeutic interventions.

## Conclusion

Patients with RA and coronary-artery atherosclerosis have higher Framingham cardiovascular risk scores even after adjustment for age and gender. However, current risk stratification classified most patients with RA as having a low 10-year risk of hard events. The use of coronary-artery calcium scores may add information to the assessment of cardiovascular risk in patients with RA and may lead to better guidelines for therapeutic interventions in this patient population. Further prospective studies are needed to examine the utility of the Framingham and the modified Framingham scores to predict hard events in patients with RA.

## Abbreviations

CI = confidence interval; DAS28 = disease activity score using 28 joint counts; HDL = high-density lipoprotein; HU = Hounsfield units; LDL = low-density lipoprotein; OR = odds ratio; RA = rheumatoid arthritis; SLE = systemic lupus erythematosus.

## Competing interests

CPC received a CHORD (Centocor Health Outcomes in Rheumatic Diseases) Fellowship in 2004 and support from the following grants: HL065082, HL067964, HS010384, and HHSA290200500421. IA received an American College of Rheumatology Research and Education Foundation 2006 Fellowship award. In PR's opinion, this is not relevant to the article, but in the interests of full disclosure, an honoraria is declared (Genzyme Corporation, Cambridge, MA, USA). TS received support from grant HL067964 and honoraria (several from different companies and foundations). CMS received support from the following grants: RO1 HL65082, HL04012, HL67964, U01 HL65962, P01 GM31304, U18 HS010384, RO1 HL081707, and a Lupus Foundation grant. In CMS's opinion, these are not relevant to the article, but in the interests of full disclosure, the following are declared:consultant fees from Bristol-Myers Squibb Company (Princeton, NJ, USA) for preparation of a series of lectures on trial design; royalty fees from the publication of textbooks of rheumatology; fees from medicolegal chart review in two cases of drug toxicity; an honorarium from the American Society of Clinical Pharmacology and Therapeutics for editing their journal; and fees from the National Institutes of Health for participating in peer review. CMS holds a patent on the use of interleukin-2 to monitor cyclosporine activity; the patent has yielded no income. All other authors declare that they have no competing interests.

## Authors' contributions

CPC performed data entry and analysis, assisted in study design, and wrote the first draft. AO collected and entered data and assisted with study design and manuscript writing. IA assisted with data entry, analysis, and manuscript writing. TG and AS performed statistical analysis and assisted in manuscript writing. PR analyzed the electron-beam computed tomography scans and assisted with study design and manuscript writing. TS and TP recruited patients and assisted with study design and manuscript writing. CMS provided the design and overall supervision of the study and performed data analysis and manuscript writing. All authors read and approved the final manuscript.

## Figures and Tables

**Table 1:** Clinical characteristics of patients with rheumatoid arthritis and control subjects.

Characteristic	Patients with early rheumatoid arthritis (*n *= 89)	Patients with long-standing rheumatoid arthritis (*n *= 66)	Control subjects (*n *= 85)	*P *value^a^
Demographic variables				
Age in years	51 (43–59)	57 (48–65)	52 (44–58)	0.002
Female gender, *n *(percentage)	57 (64%)	49 (74%)	55 (65%)	0.35
Caucasian, *n *(percentage)	81 (91%)	57 (86%)	72 (84%)	0.51
Components of the Framingham risk score^b^				
Total cholesterol (mg/dl)	182 (156–210)	188 (149–211)	195 (171–216)	0.22
Smoking, *n *(percentage)	19 (21.4%)	20 (30.3%)	8 (9.4%)	0.004
High-density lipoprotein (mg/dl)	41 (37–53)	46 (40–56)	45 (39–54)	0.16
Systolic blood pressure (mm Hg)	128 (116–139)	138 (124–150)	128 (115–136)	0.001
Framingham risk score (units)	11 (8–14)	14 (11–18)	12 (7–14)	<0.001
Ten-year risk of a cardiovascular event	2% (1%–6%)	6% (2%–11%)	2% (1%–8%)	0.006
Other cardiovascular risk factors				
Family history of coronary heart disease, *n *(percentage)	23 (26%)	19 (29%)	26 (31%)	0.78
Body mass index (kg/m^2^)	28 (25–34)	28 (24–31)	27 (25–32)	0.51
Low-density lipoprotein (mg/dl)	111 (93–135)	114 (83–135)	122 (104–145)	0.07
Lp(a) lipoprotein (mg/dl)	8.1 (2.0–24.6)	8.7 (4.0–23.2)	9.9 (4.0–32.3)	0.24
Triglycerides (mg/dl)	108 (78–141)	111 (81–172)	103 (74–135)	0.43
Homocysteine (μmol/l)	9.5 (8.1–11.4)	10.7 (8.1–12.3)	8.2 (7.1–9.6)	<0.001

^a^*P *values were calculated using Fisher's exact test for categorical variables and Wilcoxon rank sum test for continuous variables;

^b^Other than age and gender. Continuous values are presented as median (interquartile range).

**Table 2:** Spearman correlations between the Framingham risk score and clinical variables in patients with rheumatoid arthritis

	ρ	*P *value
Age (years)	0.76	<0.001
Systolic blood pressure (mm Hg)	0.60	<0.001
Diastolic blood pressure (mm Hg)	0.22	0.006
Body mass index (kg/m^2^)	-0.03	0.68
Disease duration (years)	0.29	<0.001
Pack-years of smoking	0.11	0.16
Coronary calcium score (Agatston units)	0.44	<0.001
Total cholesterol (mg/dl)	0.27	<0.001
HDL cholesterol (mg/dl)	-0.02	0.83
LDL cholesterol (mg/dl)	0.22	0.005
Triglycerides (mg/dl)	0.26	0.001
Lp(a) lipoprotein (mg/dl)	0.09	0.26
Cumulative use of steroids (grams)	0.17	0.03
Cumulative use of hydroxychloroquine (grams)	-0.05	0.53
Modified Health Assessment Questionnaire (0–3)	0.01	0.90
Disease activity score (DAS28)	0.15	0.07

Higher scores in the Modified Health Assessment Questionnaire indicate more difficulty in performing activities of daily living. Higher scores for the disease activity score using 28 joint counts (DAS28) indicate greater disease activity. HDL, high-density lipoprotein; LDL, low-density lipoprotein.

## References

[B1] Pincus T, Callahan LF (1986). Taking mortality in rheumatoid arthritis seriously–predictive markers, socioeconomic status and comorbidity. J Rheumatol.

[B2] Park YB, Ahn CW, Choi HK, Lee SH, In BH, Lee HC, Nam CM, Lee SK (2002). Atherosclerosis in rheumatoid arthritis: morphologic evidence obtained by carotid ultrasound. Arthritis Rheum.

[B3] Kumeda Y, Inaba M, Goto H, Nagata M, Henmi Y, Furumitsu Y, Ishimura E, Inui K, Yutani Y, Miki T (2002). Increased thickness of the arterial intima-media detected by ultrasonography in patients with rheumatoid arthritis. Arthritis Rheum.

[B4] Gonzalez-Juanatey C, Llorca J, Testa A, Revuelta J, Garcia-Porrua C, Gonzales-Gay MA (2003). Increased prevalence of severe subclinical atherosclerotic findings in long-term treated rheumatoid arthritis patients without clinically evident atherosclerotic disease. Medicine.

[B5] Roman MJ, Moeller E, Davis A, Paget SA, Crow MK, Lockshin MD, Sammaritano L, Devereux RB, Schwartz JE, Levine DM, Salmon JE (2006). Preclinical carotid atherosclerosis in patients with rheumatoid arthritis. Ann Intern Med.

[B6] Chung CP, Oeser A, Raggi P, Gebretsadik T, Shintani AK, Sokka T, Pincus T, Avalos I, Stein CM (2005). Increased coronary-artery atherosclerosis in rheumatoid arthritis: relationship to disease duration and cardiovascular risk factors. Arthritis Rheum.

[B7] Solomon DH, Curhan GC, Rimm EB, Cannuscio CC, Karlson EW (2004). Cardiovascular risk factors in women with and without rheumatoid arthritis. Arthritis Rheum.

[B8] Dessein PH, Joffe BI, Stanwix AE (2003). Inflammation, insulin resistance, and aberrant lipid metabolism as cardiovascular risk factors in rheumatoid arthritis. J Rheumatol.

[B9] Wilson PW, D'Agostino RB, Levy D, Belanger AM, Silbershatz H, Kannel WB (1998). Prediction of coronary heart disease using risk factor categories. Circulation.

[B10] Ford ES, Giles WH, Mokdad AH (2004). The distribution of 10-year risk for coronary heart disease among US adults: findings from the National Health and Nutrition Examination Survey III. J Am Coll Cardiol.

[B11] Mahoney LT, Burns TL, Stanford W, Thompson BH, Witt JD, Rost CA, Lauer RM (2001). Usefulness of the Framingham risk score and body mass index to predict early coronary artery calcium in young adults (Muscatine Study). Am J Cardiol.

[B12] Chung CP, Oeser A, Avalos I, Raggi P, Stein CM (2006). Cardiovascular risk scores underestimate the presence of subclinical coronary-artery atherosclerosis in women with systemic lupus erythematosus. Lupus.

[B13] Arnett FC, Edworthy SM, Bloch DA, McShane DJ, Fries JF, Cooper NS, Healey LA, Kaplan SR, Liang MH, Luthra HS (1988). The American Rheumatism Association 1987 revised criteria for the classification of rheumatoid arthritis. Arthritis Rheum.

[B14] Prevoo ML, van't Hof MA, Kuper HH, van Leeuwen MA, van de Putte LB, van Riel PL (1995). Modified disease activity scores that include twenty-eight-joint counts. Development and validation in a prospective longitudinal study of patients with rheumatoid arthritis. Arthritis Rheum.

[B15] Pincus T, Sokka T, Kautiainen H (2005). Further development of a physical function scale on a MDHAQ [corrected] for standard care of patients with rheumatic diseases. J Rheumatol.

[B16] Agatston AS, Janowitz WR, Hildner FJ, Zusmer NR, Vaimonte M, Detrano R (1990). Quantification of coronary artery calcium using ultrafast computed tomography. J Am Coll Cardiol.

[B17] Harrell F (2001). Regression Modeling Strategies.

[B18] The R Project for Statistical Computing. http://www.r-project.org/.

[B19] Esdaile JM, Abrahamowicz M, Grodzicky T, Li Y, Panaritis C, du Berger R, Cote R, Grover SA, Fortin PR, Clarke AE, Senecal JL (2001). Traditional Framingham risk factors fail to fully account for accelerated atherosclerosis in systemic lupus erythematosus. Arthritis Rheum.

[B20] O'Malley PG, Taylor AJ, Jackson JL, Doherty TM, Detrano RC (2000). Prognostic value of coronary electron-beam computed tomography for coronary heart disease events in asymptomatic populations. Am J Cardiol.

[B21] Raggi P, Cooil B, Shaw LJ, Aboulhson J, Takasu J, Budoff M, Callister TQ (2003). Progression of coronary calcium on serial electron beam tomographic scanning is greater in patients with future myocardial infarction. Am J Cardiol.

[B22] Shaw LJ, Raggi P, Schisterman E, Berman DS, Callister TQ (2003). Prognostic value of cardiac risk factors and coronary artery calcium screening for all-cause mortality. Radiology.

[B23] Becker CR, Majeed A, Crispin A, Knez A, Schoepf UJ, Boekstegers P, Steinbeck G, Reiser MF (2005). CT measurement of coronary calcium mass: impact on global cardiac risk assessment. Eur Radiol.

[B24] Schisterman EF, Whitcomb BW (2004). Coronary age as a risk factor in the modified Framingham risk score. BMC Med Imaging.

